# Effect of a 12-Week High-Calorie-Expenditure Multimodal Exercise Program on Health Indices in Women With Overweight: Protocol for a Randomized Controlled Trial

**DOI:** 10.2196/51599

**Published:** 2024-06-13

**Authors:** Mitra Abdollahi Diba, Vahid Sari Sarraf, Ramin Amirsasan, Saeid Dabbagh Nikoukheslat

**Affiliations:** 1 Department of Exercise Physiology Faculty of Physical Education and Sports Sciences University of Tabriz Tabriz Iran

**Keywords:** high-calorie expenditure, multimodal exercise, International Physical Activity Questionnaire, IPAQ, body composition, metabolic health

## Abstract

**Background:**

High-calorie-expenditure training is common among endurance athletes and is an effective strategy for weight loss. Although many training protocols include walking, running, cycling, and swimming according to a target heart rate, there is limited research on high-calorie-expenditure interventions with multimodal training programs using quantitative methods.

**Objective:**

The aims of this research protocol are to (1) develop a high-calorie-expenditure training program to cover target calorie expenditure according to the trainability of women classified as overweight (according to a BMI of 25-29.9 kg/m^2^); (2) determine the effect of high-calorie-expenditure workouts on conditioning, glycemic variables, and body composition; and (3) evaluate the implementation of the intervention and results in comparison with outcomes obtained under a standard-calorie-expenditure training program.

**Methods:**

This is a randomized controlled trial with a pretest-posttest design. Participants include 33 women with a BMI in the overweight range (25-29.9) allocated to three groups: two intervention groups and one control group. The intervention will be conducted for 12 weeks. Participants in the first group will be assigned an exercise program with high energy expenditure of approximately 3000-3500 kilocalories/week in the form of 5 sessions per week with an intensity of 50%-75% maximum oxygen rate (VO_2_ max) and 60%-80% target heart rate. The second group will be assigned an exercise program with a standard energy expenditure of approximately 1200-1500 kilocalories/week with 3 sessions per week at an intensity of 60%-75% VO_2_ max, according to The American College of Sports Medicine guideline. The effects of the multimodal training program with daily tasks will be compared to those of the standard-calorie-expenditure and control (no exercise) conditions with respect to changes in glycemic indices and body composition. Daily calories will be calculated through the International Physical Activity Questionnaire and using Nutrition 4 software.

**Results:**

Preliminary results show significant weight loss in both the high- and standard-calorie-expenditure groups (*P*=.003). Significant improvements were also found in muscle percentage (*P*=.05) and BMI (*P*=.05) for the high-calorie-expenditure group. Analyses are ongoing for glycemic indices, inflammation factors, and blood parameters.

**Conclusions:**

High-calorie-expenditure training can cause further weight loss than standard exercise, which can eventually lead to greater fat mass reduction and improvement in glycemic indices. These results demonstrate that, in some cases, it may be necessary to increase the activity of women and use multimodal exercise programs with increased volume and intensity to increase the expenditure of exercise and daily activity. We found a net effect of exercise and daily activity at the individual level, whereas the daily lifestyle and physical behaviors of the participants remained constant.

**Trial Registration:**

Iranian Registry of Clinical Trials IRCT20220202053916N1; https://tinyurl.com/c8jxfw36

**International Registered Report Identifier (IRRID):**

DERR1-10.2196/51599

## Introduction

Obesity affects more than 650 million people worldwide [1]. The prevalence of obesity is estimated to have nearly tripled since 1975; it is predicted that by 2030, 1 in 5 women and 1 in 7 men globally, equating to more than 1 billion people, will be living with obesity [2]. This increase is consistent across sex and age groups, with more than half of women of reproductive age being classified as overweight (defined by a BMI≥25 kg/m^2^) or obese (BMI≥30 kg/m^2^) [3]. Overweight and obesity are significant risk factors for many noncommunicable diseases (NCDs), including hypertension, dyslipidemia, type 2 diabetes mellitus, coronary heart disease, stroke, and some cancers [1,3]. The World Health Organization classifies risk factors for NCDs into changeable behavioral factors and metabolic risk factors [4]. Modifiable behavioral risk factors include smoking, excess salt/sodium in the diet, harmful drinking, and insufficient physical activity. Metabolic risk factors include hypertension, overweight and obesity, hyperglycemia, and hyperlipidemia [5].

Exercise has many benefits, including improving the condition and sensitivity of the body and brain, enhancing mental health, improving heart function, increasing immunity, reducing anxiety, and preventing osteoporosis. Female obesity is associated with several adverse pregnancy outcomes, including miscarriage, hypertension, preeclampsia, and diabetes [6], leading to reduced fertility, mainly due to anovulation [2]. However, other factors also vary between women with obesity and those with a body weight in the optimal range, including having a harder time conceiving, even in the case of regular menstruation. The current cultural context of valuing speed, including a tendency toward fast food, fast communication, and working within the virtual space, challenges the long-term patience of modern humans, and this desire for instant pleasure is evident in all facets of life worldwide. This issue is also emerging in terms of physical activity and exercise, in which the general public seeks to reach the desired goal in the shortest possible time; this results in increasing caloric expenditure with increased physical activities along with greater nutritional restrictions [3].

The most popular training methods at present for improving maximal oxygen consumption (VO_2_ max) are circuit training (CT) and high-intensity interval training (HIIT), which are commonly adopted by the general population, health and fitness professionals, and physiologists [7]. CT is typically performed at a moderate or high intensity over a period of 30-50 minutes and involves a range of aerobic, body weight, and resistance exercises with minimal rest [8]. Low-volume HIIT is defined as “short intermittent bursts of vigorous activity, interspersed with periods of rest or low-intensity exercise” [7], which is usually prescribed at a workout intensity of 80% to 100% of the maximum heart rate [9].

Whole-body CT and low-volume HIIT consisting of 8- to 12-minute interval episodes, interspersed with similar recovery time, have been shown to improve cardiometabolic health and cardiorespiratory fitness. The American College of Sports Medicine (ACSM) recommends that individuals perform moderate exercise 5 times per week, vigorous exercise 3 times per week, or a combination of moderate and vigorous exercise 3-5 times per week [10]. However, the optimal exercise type for improving overall health remains unclear [11]. Caloric expenditure is a common measure that can be used to compare different implementation systems. However, there are still no adequate doses for the workout tailored to specific populations [7].

The ACSM also identified the top 20 fitness trends for Australia, Brazil, Europe, Mexico, Portugal, Spain, and the United States, featuring individual fitness trends, including weight loss and unique health goals [9]. The new guidelines for adults specify a target range of 150-300 minutes of moderate-intensity physical activity and 75-150 minutes of vigorous-intensity physical activity, which differs from the previous guidelines that focused on achieving at least 150 minutes of moderate-intensity activity or 75 minutes of vigorous-intensity activity per week. This change acknowledges that there is a range of physical activity that maximizes risk reduction for health outcomes; in the case of older adults, multicomponent physical activity is recommended that focuses on functional balance and strength training to increase functional capacity and prevent falls [12].

Designing an exercise program above the standard level of calorie expenditure, especially for sedentary people, can be challenging. High-caloric exercises could act as a double-edged sword; on the one hand, a reduction of adipose tissue brings benefits of reducing metabolic syndrome, while on the other hand, high-intensity activity can be accompanied by increased inflammation. The restriction of energy intake below the amount necessary to maintain weight, known as caloric restriction (CR), can extend the lifespan even for people with normal weight. CR significantly extended lifespan in various preclinical studies with animal models; moreover, CR of approximately 30% was shown to increase life expectancy in humans by 1-5 years [13]. Thus, individuals with higher metabolic rates tend to have shorter lifetimes [14].

The development of this hypothesis stems from the inverse association observed between the rate of metabolism of mammals (by body weight and per day) and lifespan [15]. Mechanistically, the rate of life theory is supported by the free radical theory of aging [16], which postulates that 1%-3% of the oxygen consumed in mitochondrial ATP production produces reactive oxygen species (ie, oxidative stress) [17]. Therefore, with more energy expended as a function of time (ie, with aging) or because of a high metabolic rate, there is a greater likelihood of oxidative damage to the cells and tissues [18]. Oxidative stress also disrupts many molecular and cellular structures and functions. Low physical activity exacerbates this mechanism by reducing mitochondrial function and oxidation capacity in cells and tissues. Physically, caloric excess and low physical activity increase the fat mass and cause declines in skeletal muscle mass, strength, and physical fitness [19]. During a 6-month pilot study at Pennington Biomedical, participants in both CR groups lost 10% of their body weight [20] with a 25% reduction in total fat mass and a 27% reduction in visceral fat [11], the fat deposit type most closely linked to the development of metabolic diseases [16].

Similarly, participants in a study conducted at the University of Washington achieved a 20% energy deficit by exercising or losing comparable weight, total fat mass, and a visceral fat mass [13]. Although short-term CR resulted in no change in low-density lipoprotein (LDL) or high-density lipoprotein (HDL) cholesterol, 2 years of CR induced significant and persistent declines in LDL cholesterol and triglycerides, increases in HDL cholesterol, and a decreased ratio of HDL to total cholesterol; decreases in systolic and diastolic blood pressure and mean blood pressure were also observed [14]. After 1 year, CR improved left ventricular function in healthy older adults, an advantage typically observed with exercise [9]. Furthermore, the level of systemic inflammation, as measured by the concentrations of interleukin-6, tumor necrosis factor-α, and C-reactive protein, appears to have strong correlations with survival and is an important determinant of longevity in centenarians [12].

Although loss of bone mineral density is common with weight loss, regardless of the method, CR did not affect total bone mineral content in young individuals with type 2 diabetes and was deemed to be a suitable approach for weight loss in the general population [11]. However, another study showed that bone mass decreased at clinically important sites of osteoporotic fractures, such as the hip, femoral neck, and lumbar spine, after the age of 16 years [15]. These reductions were only partially proportional to changes in nutrition, physical activity, or body composition (10%-31% association in a regression analysis). This intraindividual variability in bone loss emphasizes a need for regular, close monitoring of bone health for individuals consuming a CR-based diet [16].

At present, the most popular health trend in Iran is strength training with free weights, which occupies a high proportion of the fitness industry for 2024. Specifically, eight trends related to fitness activities have emerged, including strength training with free weights, low-cost gyms, dance-based workouts, outdoor activities, Pilates, body weight training, core training, and water sports. Weight loss exercise and training methods under group-training formats ranked among the top 10 most popular health and fitness trends nationwide. There is no association between the type of training method and comfort with modern technology. Health was ranked as one of the top 20 attractive goals across the country, which is also reflected in the increased interest in aerobic sports, body weight bearing, and step training among Iranian women [17]. Furthermore, reductions in fat-free mass were associated with older age, male sex, less activity-related energy expenditure, and higher baseline BMI, and were not influenced by protein intake (normalized to body weight) [21]. Despite the greater effects of CR on fat-free mass in men, CR was found to only influence aerobic capacity and leg strength in women [19]. However, it remains unclear how specific exercise programs might affect inflammatory, oxidative, and muscle damage variables in the body in women over 35 years old who are overweight. Therefore, in this study, we aimed to evaluate whether standard- and high-calorie-expenditure exercises that are applicable to women with a BMI falling in the overweight category (ie, 25-29.9 kg/m^2^) who have not previously exercised will induce different types of changes and if there is a training capacity for 12 weeks of high-calorie-expenditure training for these women, which constitutes a form of CR.

## Methods

### Study Design

This study was designed to assess the effect of a 12-week multimodal exercise program intervention under two different calorie-expenditure conditions (high=3000 calories/week vs standard=1200 calories/week) and a no-exercise waitlist control group. The study was conducted at the University of Tabriz in the southeastern region of Azerbaijan, Iran. This study was registered in the Iranian Registry of Clinical Trials (IRCT20220202053916N1).

**Figure 1:**
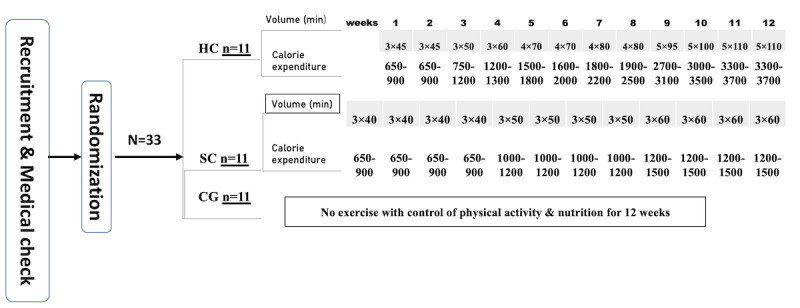
Study design and a summary of the proposed 12-week training programs for the two intervention groups and the control group. CG: control group; HC: high-calorie-expediture exercise; SC: standard-calorie-expenditure exercise.

Figure 1 summarizes the proposed 12-week training plan. Participants will be allocated to three groups: a high-calorie-expenditure group, standard-calorie-expenditure group, and control group. In the high-calorie-expenditure group, participants will engage in 3 sessions of exercise per week for 45 minutes with an estimated expenditure of 650-900 calories per week for weeks 1 and 2. In the third week, they will also have 3 sessions per week with a training duration of 50 minutes per session at an estimated calorie expenditure of 750-1200 per week. In the fourth week, the duration of the exercise will be increased to 60 minutes with 3 sessions per week, thereby increasing the calorie expenditure of the exercise by approximately 1200-1300 per week. In the fifth and sixth weeks, the training time will increase to 70 minutes and the number of training sessions will also increase to 4 sessions per week, resulting in calorie expenditure of approximately 1800-1500 and 1600-2000 calories per week, respectively. In week 7, the training duration will increase to 4 sessions of 80 minutes each with estimated weekly calorie expenditure of 1800-2200. In weeks 8 and 9, the training duration will increase to 90 minutes with expenditure of 1900-2500 calories/week. In the last 3 weeks, the number of sessions will reach 5 with weekly sessions of 95, 100, 110, 110, and 110 minutes, respectively accounting for 2700-3100, 3000-3500, and 3300-3700 calories consumed in week 10, 11, and 12, respectively.

The standard-calorie-expenditure group will receive a 12-week exercise program in the form of 3 training sessions. In weeks 1 to 4, each session will comprise 40 minutes of exercise with 650-900 calories consumed per week; in weeks 5 to 8, sessions will last 60 minutes with 1000-1200 calories consumed per week; and weeks 9 to 12 will involve an increase in the training time to 80 minutes with expenditure of 1200-1500 calories per week.

The control group will not undergo exercise training, but will undergo the same blood tests, body composition measurements, appetite measurements, and nutrition and physical activity records as performed in the other two groups.

The schedule of data collection for the main outcome measures is presented in Table 1. Primary and secondary outcomes are measured 2 weeks prior to commencing the exercise training (baseline) and 2 days after completing the 12-week exercise or control period. We are also assessing the effectiveness of different types of exercise programs in mediating the risk of metabolic syndrome, including blood pressure disorders, increased blood lipids, uncontrolled blood sugar, obesity, and insulin resistance, with the goal of gaining a better understanding of the mechanism linking exercise to metabolic control. This is assessed by validating relevant formulas and other variables such as cardiovascular fitness and functional tests. Moreover, since high-calorie-expenditure exercise is associated with a goal of weight loss, there is a possibility that such a program will induce eating disorders, which is an issue that is more common in women. Therefore, two sessions were held for the participants to familiarize themselves with recording of daily food calories with the Eating Attitude Test-26 (EAT-26) questionnaire, including questions related to diet and appetite, in the 3 groups at 3 stages: before and during the course and at the end of the course. Body composition parameters were measured in the two training groups once every 2 weeks and in the control group before, in the middle, and at the end of the intervention. Blood parameters were also measured before and after the intervention to compare appetite levels. Performance tests were conducted before, during, and after the training period, and the calories consumed and physical activity were measured on a weekly basis.

**Table 1:** Schedule of tests and measures for the primary and secondary outcomes.

Tests	Week											
	1	2	3	4	5	6	7	8	9	10	11	12
Blood sample	✓											✓
Functional test	✓					✓						✓
Appetite	✓					✓						✓
Body composition	✓		✓			✓			✓			✓
Physical activity	✓	✓	✓	✓	✓	✓	✓	✓	✓	✓	✓	✓
Nutrition	✓	✓	✓	✓	✓	✓	✓	✓	✓	✓	✓	✓

### Study Population

The program was designed for women who are classified as overweight (BMI=25-29.9 kg/m^2^) without an athletic background. Participants were recruited as a result of local media coverage related to fitness and health. To be considered eligible for this study, individuals had to be of female sex and aged between 35 and 50 years; informed written consent was required prior to the baseline measures and sample collection. The specific inclusion criteria are listed in Textbox 1.

Exclusion criteria are the onset of menopause during training, disruption of the menstruation cycle, and failure to participate in exercises in more than 30% of the sessions.

**Inclusion criteria for study participation.**

1. In good health without a history of specific diseases
  - physical diseases such as diseases and problems of the joints and bones or any type of physical and orthopedic injury to the extent that it would interfere with the implementation of exercises;
  - endocrine and metabolic diseases (eg, type 1 or type 2 diabetes, kidney and liver thyroid diseases);
  - cardiovascular diseases (eg, high blood pressure, coronary artery disease, atherosclerosis, peripheral vascular disease, heart disease);
  - mental and psychological diseases (eg, depression, schizophrenia, mania);
  - epilepsy;
  - anemia;
  - cancer;
  - any infectious and inflammatory diseases.
2. BMI of 25-29.9 kg/m^2^, leading to a classification of overweight.
3. Inactive; participated in sports/fitness activities less than 3 times a week for less than 20 minutes in the past year and did not participate in organized activities in the past year.
4. Premenopausal with a relatively regular menstrual cycle.
5. Not taking any drugs that affect heart rate, metabolism, and body weight (such as drugs related to diabetes or thyroid diseases), antidepressants, nonsteroidal anti-inflammatory drugs, reproductive hormones, supplements, and vitamins.
6. Have not consumed turmeric, caffeine, or alcohol 48 hours prior to each blood sample collection.
7. Completed and signed the consent and engagement forms as well as medical history forms.

### Ethical Considerations

The study protocol has been approved by the ethics committee of Tabriz University (code IR.TABRIZU.REC.1402026). All data will remain confidential and only the researchers have access to the data. Initial examinations were performed by a specialist doctor in compliance with ethical protocols, and all entrance tests were conducted with the permission of the participants and in a completely confidential manner. All participants provide informed consent before inclusion, and it is explained to them that they can withdraw from the project at any time. The participants are also assured that their information will remain strictly confidential with the research group and that the study data are completely anonymous.

All medical tests, counseling and educational services, familiarization classes with the calorimeter, and other services are provided to the participants completely free of charge. We have also insured our gym for 1 year and all safety points are guaranteed. In particular, given the outbreak of viruses such as COVID-19 and influenza, we implemented all health protocols and repeatedly announced health principles to all athletes using the gym. Before starting the research project, all participants were examined by the research team’s specialist and underwent cardiovascular tests such as echocardiography, blood pressure control, blood oxygen, and sports performance tests. Finally, all participants were provided with insurance in the case of exercise-related injuries by the research team for 1 year.

### Blinding

During the data collection, physical fitness and blood tests as well as anthropometric measurements will be performed in the test center, and the laboratory analysts will not have information about the group allocation of the samples or participants.

### Intervention Fidelity

There are two instructors per class who will administer the intervention in all cases. At random intervals, an independent assessor will observe all classes and monitor them for content consistency using a checklist based on the explicit components of the exercise intervention protocol. In addition, instructors will maintain a log of activities in each class and these logs will also be reviewed by the independent assessor to confirm intervention fidelity.

### Sample Size and Group Allocation

The sample size was determined by a power calculation to detect group differences in the primary outcomes. Previous randomized controlled trials of exercise interventions have demonstrated effect sizes between 0.24 and 1.17 in similar populations for cognitive performance measures following aerobic interventions. To obtain 80% statistical power with an α level of .05, using an *F*-test for three independent groups and an effect size of 0.6, the sample size required was determined to be 30 participants using G*Power version 3.1. Among 60 applicants, a total of 33 participants have been recruited after primary evaluations, including height, weight, fat percentage, waist circumference, BMI, confirmation of the activities performed in the last year, and the lack of medical restriction to participate in the exercise training.

Following written informed consent of all participants and an explanation of the risks and benefits of the research, participants were randomized into the three groups: high-calorie-expenditure (n=11), standard-calorie-expenditure (n=11), and control (n=11) groups.

### Outcome Measures

#### Baseline Measures

Before starting the training, the participants undergo a 2-week familiarization period with the physical tests and training protocol. Participants’ anthropometric characteristics such as height, weight, and body composition variables (determined at 220 Hz with the device) are measured prior to randomization.

#### Physical Activity

The baseline physical activity of the participants will be assessed with the International Physical Activity Questionnaire (IPAQ) short form (7 items) and long form (IPAQ-L, 27 items) considering a reference period of the past 7 days. The IPAQ-L is a widely used tool that queries 5 activity domains independently and provides specific details on physical activity intensity levels, differentiating between usual weekdays and weekend days by measuring sitting time [20].

#### Calorie Expenditure

Apple Watch Series 6 (Apple Inc) was used to measure the energy expenditure in each exercise session. Apple Watch is an accelerometer-based device that provides estimates of heart rate, distance traveled, calories burned, minutes of activity, and standing time. When using the associated Workout app, the Apple Watch continuously measures heart rate during exercise, and the heart rate sensor is designed to compensate for low signal levels by increasing both the light-emitting diode brightness and the sampling rate. Calories burned represent both active calories and total calories. Energy expenditure during exercise will be determined based on the VO_2_ max measurement in milliliters and body weight, and thus evaluated according to each participant’s individual characteristics [22]. The Apple Watch was selected for all monitoring periods and all types of training. Raw data are exported to Microsoft Excel via Apple Health [23]. The Apple Watch monitors physical activity by entering and updating information such as height, weight, and wrist circumference, and then outputs the energy consumption of the exercise [22]. The Apple Watch data will be validated using the following formulas of the ACSM for females [24]:

Calories/minute = [–20.4022 + (0.4472 × heart rate) – (0.1263 × body weight in kg) + (0.074 × age)]/4.184

Calories burned per minute = (METs × 3.5 × body weight in kg)/200

In this formula, METs refers to metabolic equivalents as a measurement of exercise intensity, where 1 MET=3.5 mL/kg/min based on completion of the 3-min exercise stage, and METs=8.975–0.065 (age in years) [22]. The energy expenditure during the day and night is then computed as follows [25]. First, the activities performed over 24 hours are calculated to obtain the approximate energy consumption for different physical activities in relation to the resting energy expenditure (REE) [26]. The value is presented for activity per hour for a healthy body and sleep, where REE×1 indicates general activities, REE×1.5 indicates light activities, REE×5.2 indicates moderate activities, and REE×5 and REE×7 indicate heavy activities. Second, the activity factor is multiplied by the duration of the activity. Third, the energy consumption for REE and physical activity is calculated as the average activity factor×exercise period×REE. Finally, the total energy consumption is calculated by multiplying the number obtained in the previous step, which is equivalent to the thermal effect of food.

#### Borg Rating of Perceived Exertion

Perceived exertion was measured according to the Borg Rating of Perceived Exertion scale [27] in each exercise with a score ranging from 6 to 20.

#### Maximum Oxygen

A respiratory gas analysis device, or metabolic gas analyzer device, is used to measure the maximum oxygen consumption during the physical activity on a treadmill or bicycle at different intensities. Queen’s step test was used to calculate the VO_2_ max (for females) according to the following formula [28]:

VO_2_ max (ml/kg/min) = 65.81 – [0.1847 × pulse rate beats (for 15 seconds)/min]

#### Muscle Preparation Test

Before the daily muscle preparation test, participants were guided through a 5-minute dynamic warm-up. The warm-up session began with a short walk to an indoor track or open gym. Next, a series of active movements targeting the pectoral, deltoid, and trapezius muscles was completed. Muscle tests included those indicated in the ACSM Health-Related Fitness Assessment Guide [29], such as the plank test (in seconds), the number of correct push-ups performed within 1 minute, and the wall squat test (how long the participant remains in squat position until fatigue). The participant is instructed to rate the pain intensity in their legs on a 0-10 numerical rating scale, with 0 defined as “no pain” and 10 as “the worst imaginable pain” [30].

#### Balance of Energy Expenditure and Energy Intake

Energy intake was recorded for each participant with daily energy intake obtained using the dietary analysis software Nutrition 4, version 1 [31].

#### Metabolic Syndrome

The siMS score was calculated as a measure of metabolic syndrome according to the following formula [32]:

siMS score = [(2 × waist circumference)/height] + glucose/reference value + triglycerides/reference value + systolic blood pressure/reference value – HDL/reference value

The siMS risk score was calculated as an expansion of the siMS score using the following formula:

siMS score × (age/50) × (family history of cardio- or cerebrovascular event=1.2, else=1)

#### Appetite and Nutrition

To assess subjective appetite sensations, the visual analog scale (VAS) is used. A VAS is typically composed of lines (of varying length) with words anchored at each end describing the extremes (ie, “I have never been hungrier” to “I am not hungry at all”). Participants are then asked to make a mark across the line corresponding to their feelings [33].

Nutrition attitude will be measured using the EAT-26 tool, a 26-item scale that assesses the symptoms and characteristics of eating disorders and yields an overall score. This questionnaire asks about the frequency of thinking and feeling about food, eating, and their body in disordered ways. Items are rated on a 6-point Likert scale, ranging from “always” to “never.” The three least symptomatic responses (never, rarely, and sometimes) are given a value of 0. A Cronbach α of 0.94 was reported for the EAT-26 in a sample of women [34]. A score of 20 or higher is considered to indicate the likelihood of having an eating disorder. EAT-26 has been validated by factor analysis in an Iranian population, which is based on three response options (never, rarely, and sometimes), and the Cronbach α was reported to be 0.86 [35].

### Standard-Calorie-Expenditure Training Protocol

According to the ACSM fitness guidelines [36], the standard-calorie-expenditure program includes 15 minutes of walking or running on a treadmill and 24 minutes of whole-body exercises in the first 4 weeks, followed by 25 minutes of walking and running and 39 minutes of whole-body exercises in the last 4 weeks. Specifically, this program involves 12 weeks of multimodal training starting with 3 weekly sessions of 30 minutes of compound training at a moderate intensity of 60%-75% VO_2_ max with 900 kilocalories of energy expenditure per week. By the end of the exercise period, the energy expenditure increases to 1200 kilocalories per week, with the exercise period increasing to 75 minutes with an intensity of 75% VO_2_ max. The details of the program are outlined in Table 2.

**Table 2:** Exercise protocol in the standard-calorie-expenditure group on a weekly and session basis (3 sessions/week).

Parameter			Week 1		Week 2		Week 3		Week 4		Week 5		Week 6		Week 7		Week 8		Week 9		Week 10		Week 11		Week 12
Total time (min)			120		120		120		120		155		155		155		155		190		190		190		190
EXE^a^ (min)			15		15		15		15		20		20		20		20		25		25		25		25
MHR^b^, %			65-75		70-85		65-75		70-85		65-75		70-85		65-75		70-85		65-75		70-85		65-75		70-85
**Whole-body exercises: 10 movements^c^**																									
	EXE time (min)	24		24		24		24		31.5		31.5		31.5		31.5		39		39		39		39	
	Sets (rest), seconds	3 (30)		3 (30)		3 (30)		3 (30)		3 (30)		3 (30)		3 (30)		3 (30)		3 (30)		3 (30)		3 (30)		3 (30)	
	Work (rest), seconds	30 (15)		30 (15)		30 (15)		30 (15)		40 (20)		40 (20)		40 (20)		40 (20)		50 (25)		50 (25)		50 (25)		50 (25)	
RPE^d^			13-15		14-16		13-15		1416		13-15		14-16		13-15		14-16		13-15		14-16		13-15		14-16
Calories/session			220-280		230-290		260-300		260-320		320-390		330-410		350-420		360-440		370-480		375-490		370-480		400-500
Total calories/week			650-850		700-900		780-900		800-950		920-1185		950-1200		100-1300		110-1340		115-1440		120-1470		115-1440		120-1500

^a^EXE: aerobic exercise.

^b^MHR: maximum heart rate.

^c^In the whole-body section, 10 movements are performed in 3 sets, which increases based on the addition of long-term load and training volume, including a change in resting interval intensity (active recovery and low intensity/recovery such as jogging). Based on the timing of the table, in the range between each set, there is a constant passive recovery period of 30 seconds.

^d^RPE: rate of perceived exertion; calculated as the maximal effort movement for each subject in 30 seconds based on a minimum of 6 when at rest and 20 at the maximum heart rate. During the initial session, we determined the RPE to range from 13 to 15.

### High-Calorie-Expenditure Training Protocol

Compared to the standard program, the exercise prescription for the high-calorie-expenditure group has a longer duration (45-110 vs 40-65 minutes per session), lower intensity (50%-75% vs 60%-85% VO_2_ max), and more frequent exercise (5 vs 3 times a week). Walking and running are the preferred forms of exercise to maximize calorie burning in comparison to weight training (cycling or rowing), which burns fewer calories. In the high-calorie-expenditure group, the goal of exercise expenditure is 3000-3500 kilocalories per week [30], which is achieved after 2-4 weeks of gradual lengthening of the exercise periods. The training protocol also lasts for 12 weeks, with 45 minutes of whole-body training at 65%-85% VO_2_ max consuming 1200 kilocalories per week; based on the principle of gradual overload with increasing weight, the energy expenditure at the end of the training period will be 3500 kilocalories. The calories increase per week [37] and the training time increases to 112 minutes. The main element of training is the same as that of the standard protocol, which starts with 3 sessions per week and gradually increases to 5 sessions per week, although the duration of each training session will be twice that for the standard-calorie-expenditure program in each step (Table 3).

In addition, the participants will be taught how to measure their heart rate in 10 seconds using the two index and middle fingers to find the pulse of the carotid artery of the neck and the pulse of the wrist. Participants will report their heart rate during aerobic exercise when requested by the investigator [38]. After 1 minute of active rest, each participant will begin a full-body exercise.

**Table 3:** High-calorie-expenditure training protocol on a weekly and session basis (3 sessions/week).

Parameter			Week 1		Week 2		Week 3		Week 4		Week 5		Week 6		Week 7		Week 8		Week 9		Week 10		Week 11		Week 12
Total time (min)			135		135		150		150		246		280		340		340		475		525		530		530
EXE^a^ (min)			20		20		20		30		30		30		40		40		40		50		50		50
MHR^b^, %			65-75		70-85		65-75		70-85		65-75		70-85		65-75		70-85		65-75		70-85		65-75		70-85
**Whole-body exercises: 10 movements^c^**																									
	EXE time (min)	25		25		30		30		40		40		45		45		55		55		60		60	
	Sets (rest, seconds)	3 (30)		3(30)		3(30)		3(30)		3(30)		3(30)		3(30)		3(30)		3(30)		3(30)		3(30)		3(30)	
	Work (rest), seconds	30 (15)		30 (15)		40 (20)		40 (20)		50 (25)		50 (25)		60 (30)		60 (30)		70 (35)		70 (35)		80 (40)		80 (40)	
RPE^d^			13-15		14-16		13-15		14-16		13-15		14-16		13-15		14-16		13-15		14-16		13-15		14-16
Calories/session			250-370		320-400		390-400		400-500		450-560		500-580		580-660		600-680		620-700		250-370		320-400		390-400
Total calories/week			750-1100		950-1200		1170-1200		1200-1500		1800-2000		2000-2200		2300-2600		2400-2700		3000-3200		3200-3500		3700-3900		3800-4000

^a^EXO: aerobic exercise.

^b^MHR: maximum heart rate.

^c^In the whole-body section, 10 movements are performed in 3 sets, which increases based on the addition of long-term load and training volume, including a change in resting interval intensity (active recovery and low intensity/recovery such as jogging). Based on the timing of the table, in the range between each set, there is a constant passive recovery period of 30 seconds.

^d^RPE: rate of perceived exertion; calculated as the maximal effort movement for each subject in 30 seconds based on a minimum of 6 when at rest and 20 at the maximum heart rate. During the initial session, we determined the RPE to range from 13 to 15.

### Training Exercise or Multimodal Program for One Session

**Figure 2:**
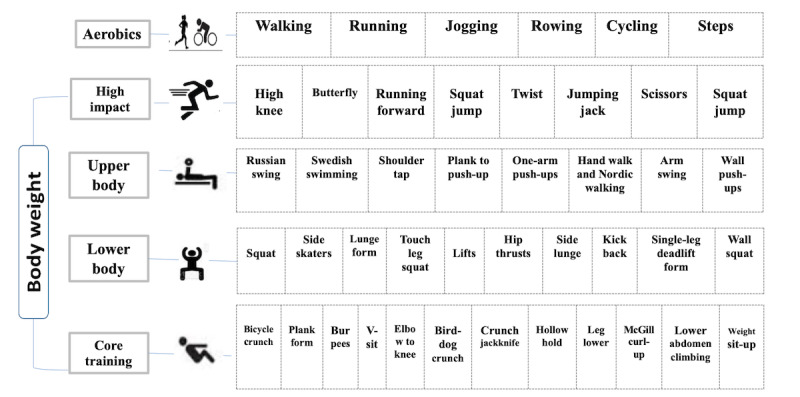
Specific exercises and drills applied in the program. In the aerobic part, participants can choose from the exercises of the first row. In the whole-body section, participants can choose 2 movements from the exercises of the 2nd row and 4 movements from the exercises of the 5th row to prevent the repetition of exercises in each session.

In both groups, participants report their weekly exercise with an exercise physiologist to estimate caloric intake and ensure compliance. After 1 minute of rest after completing the aerobic exercises, whole-body exercises are performed, consisting of 10 repetitions of the movement, in such a way as to replace the energy expenditure specified in the program and prevent repetition of the exercises. The body weight training pattern shows the energy expenditure during exercise, and the calories burned for the warm-up and cool-down periods are also calculated from the smartwatch and verified from the ACSM formula [39]. To avoid fatigue and repetition of movements during the training period, the movements are changed in a variety of ways every week, as shown in Figure 2.

## Results

**Figure 3:**
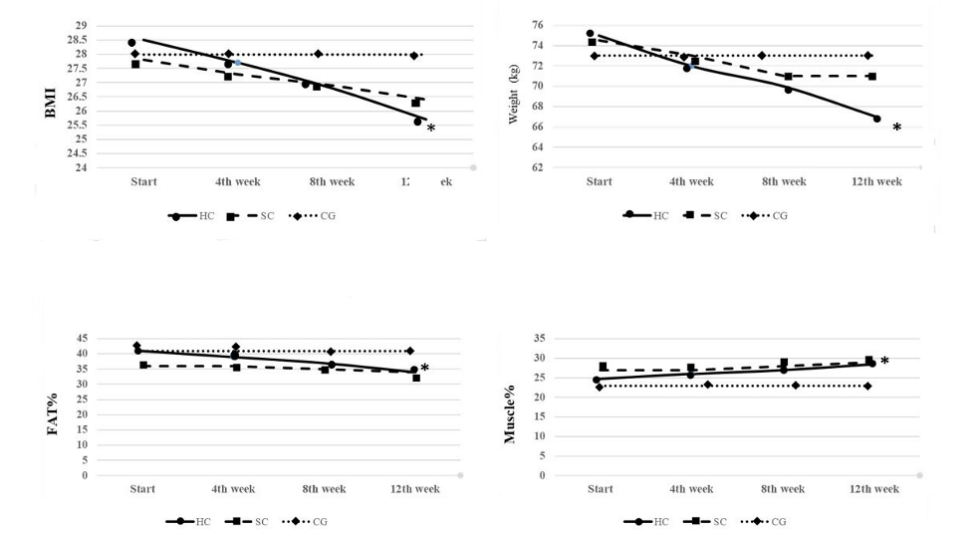
Changes in BMI, weight, fat content, and muscle in the the three groups. CG: control group (no exercise); HC: high-calorie-expenditure group; SC: standard-calorie-expenditure group.

Preliminary results show that the high-calorie-expenditure training program more than doubled weight loss and reduced fat mass, along with a further decrease in waist circumference. Figure 3 shows the changes in the body composition of the three groups. The overall goal of increasing exercise energy consumption was achieved, with an increase in energy consumption in physical activity of more than 3500 calories per week in the high-calorie-expenditure group and up to 1200 calories per week in the standard-calorie-expenditure group. Preliminary statistical analyses indicate significant differences between the stages and in the interaction of time and group. Additional analyses of functional parameters, lipid profiles, inflammatory factors, and blood indicators are ongoing.

## Discussion

### Principal Findings

The primary results of this study show that high-calorie-expenditure exercise is superior to regular exercise in terms of achieving weight loss in women with overweight. Our results demonstrate that both types of exercise (high and standard calorie expenditure) led to significant weight loss (*P*=.003). Significant changes were also observed in muscle percentage (*P*=.05) and BMI (*P*=.05) of the high-calorie-expenditure group after 12 weeks. The functional tests also showed that both exercise programs had a positive effect on the participants.

### Comparison With Prior Studies

In the case of severe and acute illnesses such as COVID-19, the effects of the disease not only decrease physical activity but also cause obesity problems in women [40]. Consequently, living an active lifestyle and monitoring one’s diet by counting daily calories can be a helpful approach to lose weight. The preliminary results of this study show that the average calorie expenditure of group-based mixed high-calorie-expenditure exercise (including the warm-up and cool-down periods) was 800 kilocalories, as estimated by the Apple Watch calorimeter. By contrast, the energy consumption per session for the standard-calorie-expenditure group was only 450 kilocalories. Results from a limited number of studies measuring energy expenditure with caloric intake have been mixed. Overall, participants performing high- and standard-calorie-expenditure exercises burned 3700 and 1350 calories, respectively. Brisebois [41] showed that 30 adults (19-60 years old, 50% women) had an average energy expenditure of 7.5 kilocalories/minute in a 60-minute CrossFit session. In a 2018 study [42], a 10-month implementation of an integrated neuromuscular circuit-type training program in small groups resulted in (1) an increase in daily energy expenditure relative to energy intake, leading to a decrease in body mass and fat; (2) an increase in strength and cardiovascular performance; and (3) a high adherence rate. Exercise-induced gains were reduced but not lost after a 5-month no-exercise period. The exercise program involved noninjury HIIT using resistance exercises of the whole body and cardiovascular activities with a limited time commitment and organized in a small group, resulting in favorable adaptation in the body and fat mass of people with overweight and obesity. Circuit-type neuromuscular exercise training protocols using only body-weight exercises could reduce body mass and fat and improve strength and endurance performance in women. These changes were attributed to an increased resting metabolic rate and lean mass, leading to increased energy expenditure. Weight/fat recovery from nonexercise was limited, suggesting that this type of exercise training may promote the long-term maintenance of weight loss in adults with overweight [42]. Willis et al [43] recorded the energy expenditure of 20 men and women during a 40-minute training session delivered by video. The findings indicated that the participants burned an average of 392 kilocalories per session (9.8 kilocalories per minute). Differences in the type and intensity of exercise throughout the session can explain the differences in the results of our study and those of previous studies. In addition, Willis et al [43] found a significant difference in energy expenditure between men and women of ~134 kilocalories per session (3 kilocalories/minute). This previous study compared the traditional exercise method with other methods. Therefore, it was not based on whole-body training and the sessions all involved steady-state exercise at the same intensity. Weight training is usually performed at high intensity for short periods with longer periods in between, which is expected. In this study, the timing of the training protocols was modified, but the protocol was similar to that of resistance training.

Monteiro et al [44] indicated that CT requires the consumption of more calories than resistance training. CT, resistance training, and combined exercises such as using a treadmill or bicycle have not been compared. Skelly et al [45] compared single sessions of two cycling protocols, HIIT and moderate steady-state endurance exercises, with a constant caloric-expenditure protocol, showing that oxygen consumption was higher in the steady-state protocol than in the HIIT protocol. However, the steady-state protocol was of a much longer duration (50 vs 20 minutes). Therefore, it can be assumed that if the duration of the two sessions were equal, the HIIT protocol would burn more calories [45]. A 6-month pilot study conducted at Pennington Biomedical Research Center [46] compared 25% CR achieved through (1) dietary restriction alone and (2) a combination of dietary restriction and exercise. Compared to the free control group, CR and exercise led to an increase in energy expenditure by 12.5%. The primary hypothesis was that CR would attenuate primary aging processes, as represented by the presence of metabolic adaptation, which is a reduction in the rate of energy expenditure beyond what would be expected on the basis of body size. In support of the hypothesis, they found that the mass-adjusted sleeping energy expenditure was reduced after 6 months of CR and CR+exercise, indicating metabolic adaptation and perhaps a delay of primary aging [46].

Among the multifaceted combined exercises highlighted above, body weight and aerobics exercises are highly valuable and can provide powerful results for middle-aged individuals. In particular, these exercises have been shown to improve cardiovascular performance and respiratory health, reduce weight, reduce body fat and increase muscle mass, improve blood circulation, improve bone and skeleton conditions, and finally improve the quality of life of middle-aged people [47,48]. In addition, in line with the prominent health-related effects of a multimodal exercise program, Batrakoulis et al [49] showed that combined aerobic and resistance exercise was the most effective method and hybrid exercise was the second most effective method for cardiometabolic health outcomes in adults with overweight and obesity, indicating higher efficacy for multicomponent exercise interventions compared to single-component methods such as continuous endurance training as a periodic exercise. Subgroup analysis of resistance training further showed that the effects of different types of training were mediated by gender [49]. This study also supports recent findings and exercise guidelines for individuals with overweight/obesity, highlighting the importance of a multicomponent exercise approach to improving cardiometabolic health. Accordingly, physicians and health care professionals should prescribe multicomponent exercise interventions for adults with overweight and obesity to maximize clinical outcomes [48]. In the present protocol, ACSM guidelines were suggested with some changes to increase the caloric expenditure of the exercise. Although HIIT exercises can also be effective, according to international guidelines for physical activity and exercise and guidelines for sedentary behavior, obesity, and type 2 diabetes, it is suggested that before starting HIIT exercise protocols, individuals should achieve a baseline level of physical fitness. Participating in a preliminary training program before using HIIT can help to reduce the risk of musculoskeletal injury while ensuring a positive training experience from the outset [50]. A gender difference in outcomes is anticipated because of variabilities in body weight and the higher rate of weight loss in men than in women.

### Limitations

This study has several limitations. The physical activity levels of the participants obtained with the IPAQ were not installed in the software and mobile phones of each participant. However, the results were determined to be satisfactory after answering the questionnaire. In addition, because this investigation started immediately after the lifting of restrictions related to the COVID-19 pandemic to conform with health protocols, it is possible that this long period of isolation had mental and psychological consequences on the participants.

When developing a target exercise interval, it is important to consider relevant variables to ensure that energy expenditure is at the level necessary for the health of all people. Further research is needed to confirm our results and determine the optimal group-based workouts that maximize caloric burn while increasing adaptation. In this study, we have confirmed the applicability of 5 high-calorie-expenditure training sessions in women over 35 years old, with benefits of weight loss, fat mass reduction, and muscle mass increase. In addition, our initial analyses suggest benefits with respect to inflammation and the lipid profile. However, the effects of these exercises on the immune system, bone conditions, reproduction, and other health facets remain unclear. It will also be valuable to examine the effect of this type of exercise over the longer term for 6 months to 1 year.

### Conclusion

Multimodal exercises with high calorie expenditure have a significant effect on body composition and glycemic and health indicators, and these exercises can be performed by people who are overweight, including those with type 2 diabetes. Our study only focused on women over 35 years of age with a sedentary lifestyle and a body weight in the overweight range. Future work could focus on the impact of this program on both men and women with obesity and overweight. In addition, tools and machines can be incorporated into the programs in future research. Several studies have shown that multimodal exercises may offer a means of repairing and recharging metabolism for middle-aged people with inactive lifestyles. Training programs using the ACSM’s minimum protocols for exercise have shown significant improvements in strength and endurance. The ACSM guidelines seem appropriate for a large proportion of the adult population who are seeking to gain muscle and lose fat. Research suggests that aerobic and total body exercise may play an important role in fitness and health, particularly with regard to body composition, glucose utilization, resting blood pressure, blood lipid profile, vascular status, and gastrointestinal transport, along with positive impacts on bone mineral density, heart function, back pain, arthritis, depression, metabolic syndrome, cardiovascular disease, and all-cause mortality. Therefore, it is recommended to include multimodal methods in weight loss and health promotion programs.

## Abbreviations

ACSM: American College of Sports Medicine
CR: calorie restriction
CT: circuit training
EAT-26: Eating Attitude Test-26
HDL: high-density lipoprotein
HIIT: high-intensity interval training
IPAQ: International Physical Activity Questionnaire
IPAQ-L: International Physical Activity Questionnaire long form
LDL: low-density lipoprotein
MET: metabolic equivalent
NCD: noncommunicable disease
REE: resting energy expenditure
VAS: visual analog scale
VO_2_ max: maximum oxygen rate
